# HPV Infection Profiles among People Living with HIV and HPV Vaccine Acceptance among Individuals with Different HIV Infection Statuses in China: A Systematic Meta-Analysis

**DOI:** 10.3390/vaccines11101614

**Published:** 2023-10-19

**Authors:** Defu Yuan, Shanshan Liu, Yangyang Liu, Fei Ouyang, Wei Ai, Lingen Shi, Xiaoyan Liu, Tao Qiu, Bei Wang, Ying Zhou

**Affiliations:** 1 Department of Epidemiology and Health Statistics, Key Laboratory of Environmental Medicine Engineering of Ministry of Education, School of Public Health, Southeast University, Nanjing 210009, China; 230239083@seu.edu.cn (D.Y.); 220223703@seu.edu.cn (S.L.); 230229009@seu.edu.cn (Y.L.); 220213961@seu.edu.cn (F.O.); 2 School of Public Health, Nanjing Medical University, Nanjing 211166, China; 2021110367@stu.njmu.edu.cn; 3 Department of HIV/STD Control and Prevention, Jiangsu Provincial Center for Disease Control and Prevention, Nanjing 210009, China; yylen2006@163.com (L.S.); liuxy@jscdc.cn (X.L.); qiutao@jscdc.cn (T.Q.)

**Keywords:** HIV, HPV infection, HPV vaccine, acceptability

## Abstract

To understand the HPV infection profiles among Chinese HIV/AIDS patients and the HPV vaccine acceptance among unvaccinated Chinese people with different HIV infection statuses after the HPV vaccine launch in China, this study searched Web of Science, PubMed, Cochrane Library, Embase, Scopus, CNKI, WANFANG, SinoMed, and VIP databases up to 23 June 2023, according to the registered protocol (CRD42023449913). A total of 58 studies were included. The results showed that the HPV infection rate among Chinese HIV/AIDS patients was 52.54% (95% CI: 42.11–62.86%) and higher in males than in females (74.55% vs. 41.04%); meanwhile, the rate was higher in the anus than in the cervix (69.22% vs. 41.71%). Although there was no statistical difference, the high-risk HPV infection rate (38.98%) was higher than low-risk HPV (23.86%), and single infections were more common (28.84%) than multiple infections (19.23%). HPV vaccine acceptance among the unvaccinated Chinese population was 59.19% (95% CI: 52.50–65.89%), and was slightly higher among HIV-infected rather than non-HIV-infected individuals (67.72% vs. 59.58%). There was a difference in acceptance among respondents from different regions. Although the difference in acceptance rate between males and females was not statistically significant (61.10% vs. 61.18%), MSM had a higher acceptance rate than non-MSM (84.28% vs. 59.05%). HPV infection is prevalent among HIV patients, demonstrating the need to increase the frequency of HPV screening for PLWH. The HPV vaccine acceptance rate is higher than that of non-HIV-infected individuals. Male acceptance is almost the same as female’s, with MSM acceptance higher than non-MSM, suggesting that using MSM, especially MSM in PLWH, as an entry point may be a practical avenue to explore to further expand the scope of HPV vaccination.

## 1. Introduction

Acquired Immunodeficiency Syndrome (AIDS) is caused by infection with the Human Immunodeficiency Virus (HIV) and is characterized by severe immunosuppression and infection. According to data reported by The Joint United Nations Programme on HIV/AIDS (UNAIDS), there were 1.5 million newly reported HIV infections in 2021, of which 49% were women and girls. Approximately 4900 young women aged 15–24 were infected with HIV each week, with People Living with HIV (PLWH) reaching 38.4 million by the end of 2021 [1]. Human Papilloma Virus (HPV) is a large group of heterogeneous viruses with similar structures, usually transmitted through sexual contact, which is classified into low-risk (LR-HPV) and high-risk (HR-HPV) types according to their carcinogenicity. LR-HPV causes benign lesions that the body’s immunity can clear, while HR-HPV is more carcinogenic and causes anogenital tumors in infected individuals [2,3]. The World Health Organization’s (WHO) report states that cervical cancer is a common cancer among women, with about 570,000 women diagnosed with cervical cancer globally in 2018 and about 311,000 women dying from the disease [4]. Almost all cervical cancer occurrences are associated with HR-HPV infections, with findings showing that HPV-16/18 and HPV-16/18/31/33/45/52/58 are responsible for approximately 70% and 90% of cervical cancer cases, respectively [5], and that HPV-16/18 is also associated with the vast majority of anal cancers in both men and women [6]. In addition, HPV-6/11 among LR-HPV is also responsible for more than 90% of genital warts [7].

Studies have confirmed that HIV infection increases the risk of HPV infection; for example, cervical HPV infection is significantly elevated in female PLWH [8], and anal HPV infection is similarly higher than in HIV-negative individuals [9]. HPV infection is also higher in male HIV patients than in HIV-negative men [10], and the HPV multi-infection (infection with more than one subtype HPV) rate in MSM is even seven times higher than in HIV-negative individuals [11]. HIV also affects HPV clearance. A prospective study monitoring cumulative HPV prevalence among women found that HPV prevalence among HIV-negative women increased from 22% to 66%, while HPV prevalence among female PLWH increased from 53% initially to 92% at the end of the study [12]. It has also been pointed out that HIV promotes the development of tumors after HPV infection. The results of a study in America showed that the incidence of anal cancer in female PLWH was 30/100,000, but no cases were found in HIV-negative women; even the risk of anal cancer in male PLWH was 80.3 times higher than that in HIV-negative males [13]. Undoubtedly, the co-infection of HIV and HPV brings a heavier disease burden for patients, but the existing results have not yet fully revealed the interaction between HIV and HPV infections. It is necessary to understand the HPV infections in PLWH first to carry out further research. A meta-analysis of 71 studies, published at the end of 2022, summarized HPV infection profiles among 856,535 Chinese women (non-HIV-infected) and showed that HPV prevalence was 14.3%; the LR-HPV and HR-HPV infection rate was 2.7% and 11.3%, respectively [14]. However, only a few studies have been conducted to summarize HPV infection profiles in a specific site (cervix or anus), in a specific PLWH group (e.g., MSM or women), in China, so there is a lack of comprehensive analyses of HPV infection in different sites in the whole group of HIV/AIDS patients.

Currently, available HPV vaccines mainly include the bivalent vaccine (Cervarix, which protects against the acquisition of HR-HPV-16 and HPV-18), quadrivalent vaccine (Gardasil, against HPV-6/11/16/18), and the 9-valent vaccine (Gardasil 9, against HPV-6/11/16/18/31/33/45/52/58); the bivalent and quadrivalent vaccines have shown promising safety and efficacy in previous research studies [15,16,17]. In addition to producing an immune response against HPV-31/33/45/52/58, girls aged 9–15 who received three doses of the 9-valent vaccine also produced an immune response against HPV-6/11/16/18, which was similar to the quadrivalent vaccine, and did not differ from the quadrivalent vaccine in terms of safety [18]. A study of the 9-valent vaccine in 14,215 cases of females aged 16–26 also obtained similar results [19], demonstrating the safety and efficacy of the 9-valent vaccine, making HPV vaccination the most effective means of preventing HPV infection. In a meta-analysis of the safety and efficacy of HPV vaccines among PLWH, the authors noted that although the evidence for the HPV vaccine effectiveness against HPV infection and related diseases is unclear for the eight available trials with low quality, the results still show that although the seropositivity of PLWH decreases over time after HPV vaccination, it can last for at least 2–4 years, and the safety is guaranteed in PLWH because HPV vaccines have caused almost no serious adverse events [20].

China’s State Food and Drug Administration (SFDA) approved the bivalent and quadrivalent HPV vaccines for marketing in July 2016 and May 2017, respectively, and then conditionally approved the 9-valent HPV vaccine for marketing in April 2018; two domestically produced bivalent vaccines have subsequently been approved. Previous surveys on HPV vaccine acceptance have mainly been conducted among age-appropriate women. One study summarized the results of several surveys conducted in China from 2009–2016 and found that the HPV vaccine acceptance among women under age 45 was 76.12% [21]. Another meta-analysis incorporating 73 studies reported the HPV vaccine acceptance among Chinese people, which was shown to be 71.8% [22]. However, most of the surveys included in these studies were conducted before the HPV vaccine was available in China, and a survey of HPV vaccination in China conducted at the end of 2021 found that only 24.39% of women surveyed had received the HPV vaccine [23]. None of the previous review studies restricted the HPV vaccination history and HIV infection status of the respondents, and few studies reviewed the acceptance profiles after the launch of the HPV vaccine. There are also fewer surveys of HPV vaccine acceptance among PLWH in China. Most HPV-vaccine-acceptance studies did not differentiate between survey respondents concerning their HPV vaccination history, so the acceptance among the unvaccinated population is still unknown in China.

In this study, we hope to understand the HPV infection profiles among PLWH by summarizing the results of related studies and further analyzing them according to different genders, infection sites, HPV types, and other characteristics to find out the differences in HPV infection profiles among different subgroups of PLWH. Meanwhile, we will comprehensively analyze the HPV vaccine acceptance level among unvaccinated people after the launch of the HPV vaccine in China, and analyze the specific situations among individuals with different HIV infection statuses, gender, and other characteristics, not only to provide reference information for accurately grasping the actual acceptance of the HPV vaccine among unvaccinated women who are still in a wait-and-see state in China, but also to explore possible entry points for further expanding the scope of HPV vaccination in the future, especially in its application for males or PLWH.

## 2. Materials and Methods

### 2.1. Search Strategy

The review protocol was registered on PROSPERO (CRD42023449913). The databases Web of Science, PubMed, Cochrane Library, Embase, Scopus, CNKI, WANFANG, SinoMed, and VIP served as the study’s data sources. Search terms included HIV, AIDS, HPV, Human Immunodeficiency Virus, Acquired Immune Deficiency Syndrome, Human Papillomavirus, Acceptability, Willingness, Attitude, Intention, and China. Before the formal search, all the search terms were also searched in the Chinese Theme Word List and Mesh Word List, and several commonly used expressions were added. An additional manual retrieval strategy was used to retrieve and supplement the related literature that was listed in the review. Without regard to the article type, the retrieval time was from the earliest year provided by the database to 23 June 2023.

### 2.2. Inclusion and Exclusion Criteria

#### 2.2.1. Prevalence of HPV Infection among PLWH in China

Inclusion criteria: (1) The literature was a cross-sectional observational study, published in English or Chinese before 23 June 2023. (2) The study population was Chinese PLWH who underwent HPV testing. (3) The investigation was conducted in mainland China. (4) The article reported the accurate number of patients included in the study as well as HPV detection. (5) The sample size was not less than 100.

Exclusion criteria: (1) Non-original studies (review, meta). (2) Studies limited to patients with obvious symptoms of HPV infection. (3) Cohort studies with therapeutic interventions for patients.

#### 2.2.2. HPV Vaccine Acceptance in Unvaccinated Chinese People with Different HIV Infection Statuses

Because men have not yet been included in the HPV vaccination program in China, only women were limited by prior vaccination history; for example, the survey respondents had to be explicitly unvaccinated women, or the acceptance of unvaccinated women was reported separately in the results section. However, if the text did not explicitly describe that the men included in the survey were previously vaccinated with the HPV vaccine, the default was that the men had not been vaccinated against HPV.

Inclusion criteria: (1) The literature was of cross-sectional observational studies published in English or Chinese before 23 June 2023. (2) The study population was Chinese people (who had not received the HPV vaccine) who were surveyed on HPV vaccine acceptance. (3) The survey was conducted in mainland China. (4) The survey was conducted after July 2016. (5) The article explicitly reported the number of unvaccinated patients as well as the number of people who were willing to receive the HPV vaccine.

Exclusion criteria: (1) Non-original studies (review, meta). (2) Studies which measured the respondents’ willingness to vaccinate others (spouses/children) with the HPV vaccine. (3) Studies in which educational interventions with relevant knowledge were provided to the patients. (4) Studies carried out under the condition of assuming a variety of prerequisites (the vaccine is provided free of charge, the vaccine is completely free of side effects, etc.).

If different articles were reported using the same data, only the article with the most detailed data description was included.

### 2.3. Literature Screening, Data Extraction, and Quality Assessment

The software Endnote (version X9) is used to manage the retrieved bibliographies. Data will be extracted independently by two writers. Any disagreements will be settled through debate until an agreement is reached or a third author is consulted. The following information will be extracted: the first author, the year of publication, the title (Chinese and English), the sample source, the investigation time, the collection sample, the study population, the number of HIV patients, the HPV infection profiles, the number of distributed and valid questionnaires, the number of unvaccinated and willingly vaccinated individuals, etc. If they included subjects with different affected areas or genders in one study and accurate data were available, they were extracted for multiple data points. The STROME-ID statement and features of the related studies were considered when we modified the literature-quality-assessment items, following the AHRQ’s requirements for evaluating cross-sectional studies. A total of 11 assessment items were eventually set for each component (Supplementary Material File S1).

### 2.4. Subgroup Designs

Publication Language: Chinese; English. Gender: Male; Female. HPV type: High-risk HPV; Low-risk HPV. Infection type: Multiple-infection (MI); Single-infection (SI). HIV infection statuses: HIV infection; Non-HIV infection. Sample source: North China (Beijing, Tianjin, Hebei, Shanxi, and Inner Mongolia), Northeast (Heilongjiang, Jilin, and Liaoning), East China (Shanghai, Zhejiang, Jiangsu, Anhui, Jiangxi, Shandong, and Fujian), Central China (Hunan, Hubei, and Henan), South China (Guangdong, Guangxi, and Hainan), Southwest (Yunnan, Guizhou, Sichuan, Chongqing, and Tibet), Northwest (Xinjiang, Gansu, Ningxia, Qinghai, and Shaanxi). Multi-region (sample sources include two or more provinces/autonomous regions/municipalities). Meanwhile, the HPV vaccination policies in Hong Kong, Macao, and Taiwan differ from mainland China; studies about those regions are also excluded.

### 2.5. Statistical Analysis of Data

Software R (version 4.2.3) and Stata (version 16.0) were used for the statistical analysis. The meta package evaluated the combined effect value of the HPV infection rate among PLWH and the HPV vaccine acceptance among unvaccinated Chinese people. The heterogeneity test employed the Q test and I^2^ test. When *p* ≤ 0.10 or I^2^ ≥ 25%, it indicated study heterogeneity. The combined effect size was determined using the random-effects model when *p* ≤ 0.10 or I^2^ ≥ 50%; otherwise, the fixed-effects model was used. Subgroup analyses and meta-regression analyses were also conducted. For the investigation of publication bias, Egger’s test was employed. If the *p* value > 0.05, it was assumed that there was no substantial publishing bias; if there was, the clipping and complement approach was employed to rectify it. Sensitivity analyses were also conducted by eliminating studies one by one to evaluate the stability of the combined effect values.

In this study, the HPV infection rate among PLWH was the number of HIV/AIDS patients infected with HPV (without distinguishing the type of HPV) divided by the number of PLWH who were successfully tested for HPV, hereinafter referred to as the infection rate; the HR-HPV infection rate, LR-HPV infection rate, single-infection rate, and multi-infection rate were similar. HPV vaccine acceptance among unvaccinated Chinese people is the number of people willing to receive the HPV vaccine as a proportion of the unvaccinated population who completed a valid survey, hereinafter referred to as acceptance; male acceptance and female acceptance are similar.

## 3. Results

### 3.1. Literature Screening Process

**Figure 1:**
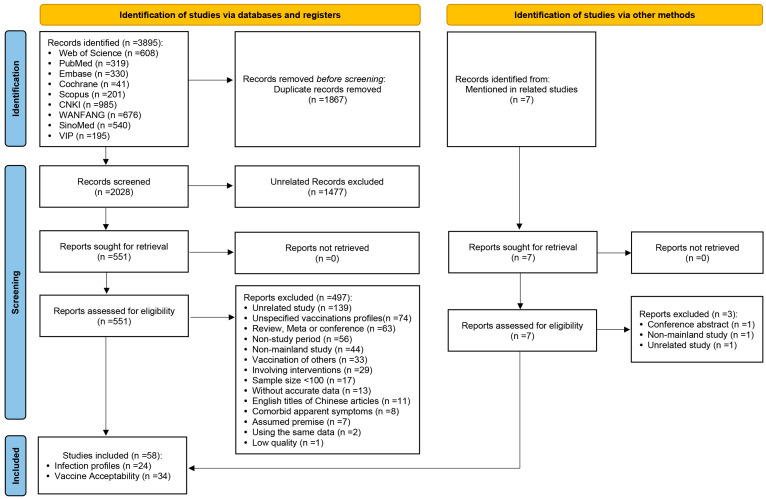
PRISMA 2020 study-selection flow diagram for the meta-analysis of HPV infection profiles in PLWH and HPV vaccine acceptability among unvaccinated Chinese.

Figure 1 [24] illustrates the process of literature screening. A total of 3895 references were obtained from the database searched in this study. After eliminating 1867 duplicates, the titles and abstracts of the remaining 2028 documents were read, and 1477 documents unrelated to the topic of this study were again removed. The remaining 551 articles were read in full. After reading the full text, 497 papers that did not meet the inclusion criteria were excluded (139 studies with content not directly related to the topic of this study; 74 studies did not report on the HPV vaccination history of the enrolled subjects or reported on the acceptance of both vaccinated and unvaccinated respondents in a mixed manner and were indistinguishable from one another; 63 studies were reviews, meta-analyses, or abstracts of conferences; 56 studies’ survey time was before the HPV vaccine was launched in China; 44 studies were conducted in Hong Kong, Macao, and Taiwan; 33 studies investigated the HPV vaccination acceptance for other people such as spouses or daughters; 29 studies involved interventions for the study participants, and they were the researches of the impact of interventions on the relevant conditions; 17 surveys of HPV infection among HIV/AIDS patients had sample sizes of less than 100 cases; 13 studies did not provide detailed data; 11 studies were English-language transcripts of Chinese literature; 8 studies were studies of PLWH with combined symptoms of significant HPV infection; 7 studies were surveys of HPV vaccine acceptance among participants assuming a variety of prerequisites; 2 studies used duplicated data with other published studies; and 1 study was low quality); the remaining 54 studies were included in the analysis. In addition, when reading the articles obtained from the search, seven articles were not included in the search results. However, they were mentioned by the authors as being related to the topic of this study. After searching and reading the full text in detail, three of these articles were excluded, and the remaining four were included in this study, making the final inclusion of analyzed literature 58 articles, of which 24 were surveys related to the HPV infection profiles of PLWH [25,26,27,28,29,30,31,32,33,34,35,36,37,38,39,40,41,42,43,44,45,46,47,48], and 34 were surveys of the HPV vaccine acceptance among unvaccinated Chinese [49,50,51,52,53,54,55,56,57,58,59,60,61,62,63,64,65,66,67,68,69,70,71,72,73,74,75,76,77,78,79,80,81,82].

### 3.2. Characteristics of Studies on the HPV Infection Profiles among PLWH in China

A total of 24 relevant studies were included in this section. Because one study collected samples from both the cervix and the anus of patients and reported the results separately, the study was extracted into two data points, resulting in a total of 25 data points, among which 17 studies (18 data points) reported infection profiles without differentiating between the types of HPV, and 7 studies reported only high-risk or low-risk HPV infection.

Table 1 lists the characteristics of the 24 included studies (25 data points), which were mainly conducted in Yunnan, Guangdong, Zhejiang, Hubei, and Guangxi provinces, and involved a total of 6720 HIV/AIDS patients, including female PLWH, male PLWH, and MSM in PLWH, with cervical (cervical exfoliative cells) and anal (anal swabs) sampling sites. Studies were grouped as high, except four which were grouped as the medium group, in quality scores (Supplementary Material File S2).

### 3.3. Meta-Analysis, Subgroup Analysis, and Meta-Regression Analysis of the HPV Infection Rate among PLWH in China

**Figure 2:**
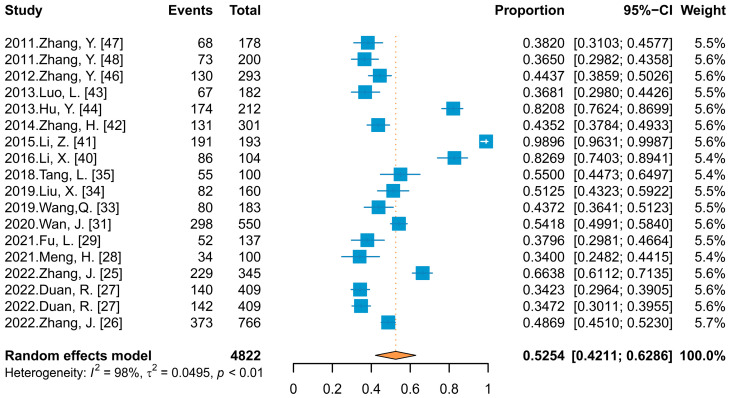
Forest plot of meta-analysis for HPV infection rate among PLWH.

In calculating the combined effect value for HPV infection rate in Chinese PLWH, data from 17 studies (18 data points) that did not differentiate between HPV types were included, in which the reported infection rate ranged from 34.00% to 98.96%, and the meta-analysis (Figure 2) showed a combined effect value of 52.54% (95% Confidence Interval (CI): 42.11–62.86%). The subgroup analysis and meta-regression (Table 2) showed that there was no statistically significant difference in the infection rate among PLWH reported in different published languages (*p* > 0.05) but there was in different genders and sampling site groups (*p* ≤ 0.05).

The combined infection rate among male PLWH was 74.55%, which was higher than among female PLWH (41.04%), and the detection rate of HPV was also higher in samples collected from the anus rather than the cervix of women (69.22% vs. 41.71%). When focusing on infection profiles in male PLWH, the results showed a higher HPV infection prevalence in MSM than in the general male population (84.91% vs. 49.13%, Q_B_ = 13.40, *p* ≤ 0.05).

### 3.4. The Infection Rates of Different HPV Types and Infection Type among PLWH in China

**Figure 3:**
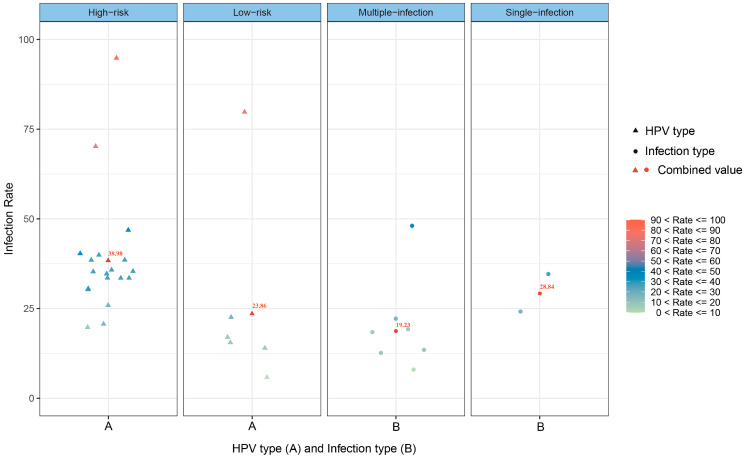
Scatterplot of the infection rates of different HPV types and infection types among PLWH in China.

Figure 3 shows the infection rate of different HPV types and infection types among PLWH in China. Of the 24 included studies (25 data points), 18 studies (19 data points) and 6 studies reported on high-risk and low-risk HPV infections, respectively, and 7 studies and 2 studies reported on multiple and single infections, respectively.

Although the differences between groups of different HPV types and infection types were not statistically significant (*p* > 0.05), the HR-HPV infection rate was 38.98%, which was higher than that of LR-HPV (23.86%), and the single-infection rate was higher than the multiple-infection rate (28.84% vs. 19.23%).

### 3.5. Characteristics of HPV-Vaccine-Acceptance Studies in Unvaccinated Chinese People with Different HIV Infection Status

A total of 34 (Table 3) cross-sectional observational surveys conducted in mainland China were included in this section, with online-virtual and offline-physical questionnaires as the primary survey forms. Nine studies involved multiple provinces or regions, and the remaining 25 were single-regional studies, resulting in a total enrollment of 57,133 cases of unvaccinated Chinese people from 31 provincial-level administrative regions in mainland China. Twenty-seven studies were limited to a single gender (male or female only), and five studies did not limit the gender but provided separate results for males and females at reporting; the remaining two did not limit the gender and did not provide separate results for different genders. More information can be found in Supplementary Material File S2.

### 3.6. Meta-Analysis and Subgroup Analysis of HPV Vaccine Acceptance in Unvaccinated Chinese People with Different HIV Infection Status

**Figure 4:**
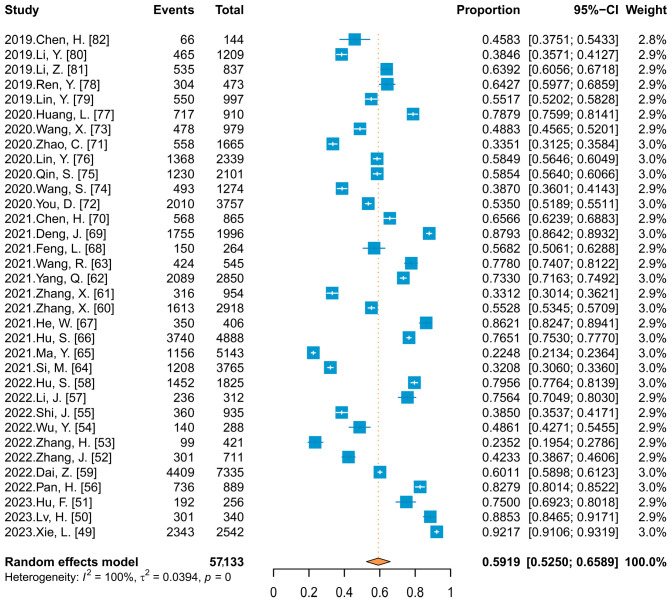
Forest plot of meta-analysis for HPV vaccine acceptability among unvaccinated Chinese people.

Thirty-four studies reported HPV vaccine acceptance ranging from 22.48% to 92.17%. Meta-analysis (Figure 4) showed that the combined acceptance was 59.19% (95% CI: 52.50–65.89%). When subgroup analyses were conducted with publication language and HIV infection status as variables, there were no statistical differences, with Chinese- and English-language published studies reporting almost identical acceptance (59.63% vs. 60.83%), and 67.72% and 59.58% among HIV and HIV-negative individuals, respectively.

### 3.7. Analysis of the HPV Vaccine Acceptance by Region in China

**Figure 5:**
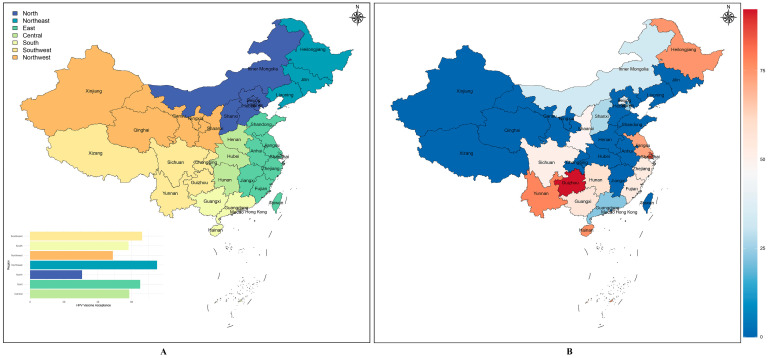
HPV vaccine acceptability among unvaccinated Chinese people in different geographical divisions (**A**) and Provincial Administrative Regions (**B**).

When divided by the seven major geographic subregions of China (Figure 5A), the HPV vaccine acceptance was different (Q_B_ = 123.02, *p* < 0.05), with Northeast China having a higher level of acceptance (75.00%) than the other regions, followed by Southwest China and East China, with 66.11% and 64.95%, respectively, and North China having the lowest level of acceptance (30.68%). One study investigated acceptance in Shanxi and Sichuan and reported the data separately of the nine studies involving multiple regions, which offer two data points, with the remaining 25 studies conducted within a single province, constituting a total of 27 data points. Figure 5B provides information on the combined acceptance for the 16 provinces, with the top five acceptance regions being Guizhou, Shanghai, Yunnan, Hainan, and Heilongjiang.

### 3.8. Analysis of the HPV Vaccine Acceptance among Different Genders in China

**Figure 6:**
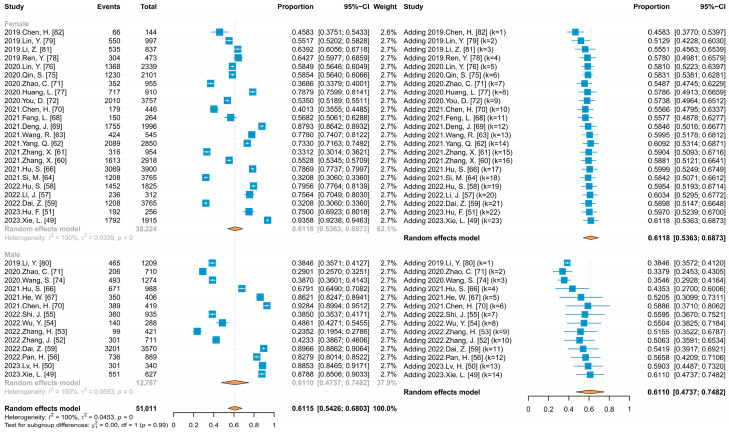
Forest plots of meta and cumulative meta for HPV vaccine acceptance by gender.

Five studies that did not limit the initial respondents’ gender provided HPV vaccine acceptance rates for males and females separately at the time of reporting, with 27 other studies that limited the respondents to a single gender, providing 37 data points. Meta-analysis (Figure 6) and subgroup analysis results showed that there was no statistically significant difference in acceptance between males and females (61.10% vs. 61.18%, *p* > 0.05), and further comparison of acceptance among men between the MSM and the non-MSM rates revealed that acceptance was significantly higher among MSM (84.28% vs. 59.05%). When the cumulative meta-analysis was performed separately by gender, using the publication time of the included studies (Figure 6), the results showed an upward trend in acceptance among females (Coef = 0.00262, *p* < 0.05), which slowly increased from 45.83% reported in 2019 and continued to fluctuate around 60%, and showed an upward trend among males (Coef = 0.01595, *p* < 0.05), increasing from 38.46% to 61.10%.

### 3.9. Publication Bias and Sensitivity Analysis

Egger’s test showed no publication bias in both components (P_1_ = 0.5186; P_2_ = 0.5322). The sensitivity analysis results showed that the maximum change value of the combined infection rate was 3.86%, when removing a study published in 2015 conducted in the Shaanxi MSM population, and the change amount was within 2.00% when excluding other studies. The maximum change value of combined acceptance was 1.12%, when excluding a study published in 2021 among students in Guangdong province, and the change amount was less than 1.12%, after removing other studies, suggesting that the combined effect values of both components were relatively stable.

## 4. Discussion

In this study, we reviewed the HPV infection profiles among PLWH in China, and the results showed that the combined HPV infection rate was 52.54%, with HR-HPV and LR-HPV infection rates of 38.98% and 23.86%, respectively, which was much higher than the infection rate of 14.3% (HR-HPV: 11.3%; LR-HPV: 2.7%) in HIV-negative women in previous studies [14]. HIV mainly invades the immune system of the human body, including CD4+ T lymphocytes and dendritic cells, which leads to a progressive decrease in the number of lymphocytes, resulting in immune deficiencies and a weakened ability to clear the virus, causing opportunistic infections and the development of tumors such as cervical cancer [83]. In addition, because HIV and HPV have the same transmission pathway, PLWH often have frequent high-risk sexual behavior, more sexual partners, and a younger age at first sexual intercourse, which also increases the chance of HPV infection. Meanwhile, HPV is known to be the main factor leading to cervical precancerous lesions and cervical cancer. If the body’s immune function is not good enough to inhibit or automatically clear the HPV infection, or the coexistence of other predisposing factors, HR-HPV persistent infection will lead to chromosome destabilization in the host cells and ultimately cause the endothelial cells of the cervical epithelium to malignantly transform [84]. Bao’s research has found that the HPV-DNA can be detected in more than 90% of cervical cancer tissues [85]. Liu et al. also showed that more patients with cervical intraepithelial neoplasia (CIN) were infected with HPV in PLWH than in HIV-negative individuals [86]. The proportion of HR-HPV infected PLWH with a CD4+ T-lymphocyte count of more than 500 (cells/μL), and a CD4+/CD8+ ratio of more than 1 were lower than those of HR-HPV-negative PLWH, suggesting that increasing the frequency of HPV screening in PLWH and initiating treatment as early as possible may contribute to reducing the disease burden, improving quality of life, and prolonging survival cycles.

Two meta-analyses in China, with search times ending in April and June 2020, have reported the HPV infection rates of male and female PLWH in China, which were 85.1% [87] and 45.8% [88], respectively, and the infection rates of male PLWH in this study were similarly higher than those of females (74.55% vs. 41.04%). Meanwhile, the HPV infection rates obtained in this study were lower than those in previous studies for both men and women, which may be related to the fact that the HPV vaccine has been approved for marketing in China, and some women have been vaccinated with the HPV vaccine, which has produced antibodies against HPV and reduced the infection rate in women. Since HPV is mainly transmitted through sexual contact, reducing HPV infection in women may also reduce the risk of infection in the men who encounter them. Previous studies have demonstrated the safety, immunogenicity, and efficacy of HPV vaccines in immunocompetent young people. Although some studies have considered that PLWH may not achieve the expected results after receiving many conventional vaccines due to their compromised immune systems [89]; for example, about 20–70% of PLWH have no immune response after the hepatitis B vaccine, the safety and efficacy of bivalent and quadrivalent HPV vaccines in PLWH have been verified. A female PLWH study [90], showed that PLWH with the bivalent vaccine had lower levels of HPV16/18 geometric mean titers (GMTs) of neutralizing antibodies compared with HIV-negative individuals but were still somewhat protective compared with the placebo. Furthermore, HPV vaccination did not affect the CD4+ T-cell counts or HIV viral load of PLWH and had no serious adverse effects. Studies related to the quadrivalent vaccine have found similar findings in 7–12-year-old children [91] and men aged 27–45 [92]. In PLWH between 13 and 27 years old with CD4+ T-cell counts of more than 350 (cells/mm^3^), also reported were no significant differences in HPV IgG concentrations between the PLWH and HIV-negative individuals over time [93]. Additional findings have shown that female PLWH receiving ART have twice as many GMTs as non-ART patients [94], and male PLWH receiving ART only need a CD4+ T-cell count of more than 200 (cells/μL) for better immunogenicity, but the non-ART male PLWH need to fulfill the requirement of more than 350 (cells/μL) [95]. These results all support the feasibility of the HPV vaccine in PLWH. The HPV vaccination program among women of appropriate age in mainland China has improved HPV infection profiles among women, and severe HPV infection among male PLWH may also be suppressed by applying the HPV vaccine in the future. However, more studies on the safety and efficacy of the HPV vaccine are required, especially on the 9-valent vaccine in PLWH.

Although single-infection is more common when grouped by the number of HPV subtypes infected, the pooled multi-infection rate is also nearly 20%. HIV infection makes it difficult for patients with impaired immune function to clear infected HPV automatically. As the organism continues to be infected with HPV, the degree of the lesion at the infection site will continue to deepen, and the likelihood of multi-infection will also continue to rise as the lesion’s severity increases, which in turn exacerbates the malignant lesion. Some studies have concluded that ART for HIV fails to affect HPV infection rates [96], but this may be because ART prolongs the survival time of PLWH without significantly altering the high-risk behavioral habits, resulting in no decrease or an increase in the risk of HPV infection. More studies have pointed out that ART can help PLWH achieve varying degrees of immune reconstruction, and reduce the probability of HPV infection risk and persistent infection, such as a 9% reduction in the monthly risk of HPV infection among patients receiving ART [97], a significant reduction in anal HPV infection risk among MSM in PLWH receiving ART [98], and a nearly half reduction in the risk of HPV infection and cervical lesions among female PLWH receiving ART in South Africa [99]. This suggests that PLWH should maintain regular ART on top of increasing the frequency of HPV testing for timely detection and early treatment of HPV infections, as it contributes to the enhancement of immunity, promotion of HPV viral clearance, reduction of multi subtype HPV co-infections, and even controlling the development of malignant lesions.

This study summarized data from studies that focused only on the unvaccinated Chinese population and studies that mixed the HPV-vaccinated population but provided separate information on the acceptance of the unvaccinated individuals, and the results showed that the HPV vaccine acceptance among unvaccinated Chinese people was 59.19% after the HPV vaccine was permitted in China. Except for a few individuals, the majority of Chinese people in mainland China had not been HPV vaccinated prior to the marketing permission. However, the acceptance obtained from pre-permission studies was still higher than this study [21,22], which is most likely related to the fact that some of the surveys at that time set many assumed preconditions, such as being provided free of charge, having no side-effects at all, or having perfect protection. Whereas, the data included in this study were only investigated based on the results of the survey respondents’ answers on their knowledge of HPV and its vaccine and did not set any preconditions, so some of the survey respondents chose not to be vaccinated, taking into account factors such as the high cost, the presence of side effects, and the lack of lifelong efficacy [49,56,59]. Previous studies in China noted that individuals with more knowledge about HPV were 1.796 times more likely to receive the HPV vaccine than those with less knowledge [49]; a higher proportion of individuals who perceived themselves to be at higher risk of HPV infection were willing to receive the HPV vaccine compared to those at lower risk (97.5% vs. 80.1%) [67]; and compared to individuals without sexual intercourse history, individuals with sexual intercourse history, both male and female, were 1.558 and 1.646 times more receptive, respectively [59]. The proportion of individuals with STD history who were willing to receive the HPV vaccine was also significantly higher than that of individuals who had not been diagnosed with an STD (92.16% vs. 81.58%) [56], which means that the survey respondents’ knowledge of HPV-related diseases, HPV and HPV vaccine, the level of perceived HPV infection risk, the sexual behavior history, and the STD history all influence the individual’s attitude toward the HPV vaccine. PLWH are characterized by frequent high-risk sexual behaviors, multiple sexual partners, and high rates of STD, and the factors mentioned above may also be partly responsible for the higher acceptance from PLWH than non-HIV individuals, suggesting that appropriately adjusting the price of the vaccine, strengthening the publicity and promotion of related knowledge, especially the safety and efficacy of the HPV vaccine, and increasing awareness and knowledge of HPV and its vaccine will be conducive to increasing the HPV vaccine acceptance and vaccination rate.

It is worth mentioning that although there was no difference in acceptance between males and females, according to the results of cumulative meta, both unvaccinated males and females had an increase in HPV vaccine acceptance after the HPV vaccine was permitted in China, reflecting a series of measures that have been taken, since the HPV vaccine has been approved and marketed, one after the other in China since the start of 2016, such as increased publicity of HPV vaccine-related knowledge, increase in the types of vaccines that can be administered, expansion of the age range that can be vaccinated, and lowering of the cost of the vaccine, have achieved good results. These measures have been effective among females and impacted the HPV vaccine acceptance among males. China currently only vaccinates females against HPV, and the United Kingdom has provided HPV vaccine for girls aged 12–13 years old since 2008 to prevent cervical cancer caused by HPV infection and has provided a catch-up vaccination program for females aged 14–18 years old from 2008–2010. On the one hand, this female-only vaccination program has resulted in a significant reduction in the incidence of cervical cancer and cervical intraepithelial neoplasia type 3 (CIN3) in young women and has even almost succeeded in eliminating the incidence of cervical cancer in females born since 1 September 1995 [100]. On the other hand, it has also achieved “herd protection” for unvaccinated males of the appropriate age, resulting in a significant decline in HPV infection rates among unvaccinated heterosexual males, but the HPV infection rate in the MSM remains relatively stable [101]. On this basis, as evidence emerged of an association between HPV and genital warts and non-cervical cancers, a pilot HPV vaccination program for gay and bisexual men under 45 years old in sexual health clinics began in the UK in 2016, but this program did not take oral cancer in heterosexual men into account [102]. Subsequently, the UK has offered the HPV vaccine to adolescent boys of all sexual orientations since 2019 and has contributed significantly to controlling HPV infections among men. This study found that the acceptance of MSM was much higher than that of non-MSM, reaching 84.28%, suggesting that in the process of HPV vaccine promotion in China, in addition to broadening the age range among females and providing it free of charge under certain conditions in some provinces, it might also be possible to try to use MSM or MSM in PLWH as an entry point to gradually apply it among males, which would contribute to the control of HPV infections in males and reduce the burden of disease. It would also provide some assistance in realizing the “Global strategy to accelerate the elimination of cervical cancer as a public health problem” proposed by the WHO.

To the best of our knowledge, this study is the first meta-analysis in China to include both male and female HIV/AIDS patients and to provide a comprehensive description of the infection profiles with different sampling sites, HPV species, and HPV subtypes. At the same time, we found that a large number of previous HPV vaccine acceptance surveys conducted in China, or even meta-analysis within a specific population, did not differentiate between patients’ previous HPV vaccination status and reported the acceptance of individuals with different vaccination histories in a mixed manner, which may have resulted in an overestimation of the acceptance from the unvaccinated population. Therefore, this study focused on unvaccinated individuals and compared the acceptance of individuals with different HIV statuses for the first time in China, providing more accurate reference information for further promoting the HPV vaccine in the unvaccinated population. There are also some limitations. Firstly, the heterogeneity is high, as the data of this study are all derived from published articles, much information about the study individuals’ characteristics could not be extracted. Although the subgroup analysis has found that there are differences in HPV infection profiles among PLWH of different genders and sampling sites, and also found that there are differences in HPV vaccine acceptance among individuals in different regions and with different behavioral characteristics among the same gender, but this is not enough to explain all the heterogeneity. Different HPV testing methods and tested-HPV-subtype numbers may impact the combined value. Meanwhile, this study did not include studies with sample sizes less than 100, due to concerns of the limitations of sample sizes in previous meta-analyses, which may also provide certain information, but this study could not analyze those factors in depth. More factors contributing to heterogeneity need to be further explored in subsequent studies. Secondly, although some of the studies involved respondents from multiple regions, the distribution of the source regions ultimately included in this study was not sufficiently balanced, and there are still missing data from some provinces, which may make the results of the study deviate from the actual situation to a certain extent. Thirdly, some of the subgroups included fewer study cases, which may also have an impact on the results of the relevant analysis. Finally, due to the lack of qualification of specific HPV subtypes in many studies, the extracted information about HPV subtypes is limited, whether in the infection rate or acceptance, so this study was unable to carry out a detailed analysis for a particular HPV subtype, and there is a need to continue to track the literature to analyze the effect of different HPV subtypes on these components in subsequent studies.

## 5. Conclusions

The HPV infection profiles among Chinese HIV/AIDS patients are prominent, with a significantly higher infection rate in men than in women, and more HR-HPV infections than LR-HPV, suggesting that increasing the HPV screening frequency for PLWH and initiating treatment as early as possible may help reduce the disease burden, improve quality of life, and prolong survival cycles. Meanwhile, the HPV vaccine acceptance among PLWH is higher than the non-PLWH; the HPV vaccine acceptance among unvaccinated males was almost equal to that of females and was higher among MSM than non-MSM, suggesting that using MSM, especially MSM in PLWH, as an entry point may be a practical attempt to explore and further expand the scope of HPV vaccination.

## Figures and Tables

**Table 1:** The characters of 24 studies (25 data points) of HPV infection profiles in PLWH.

ID	Year	First Author	Sample Source	Sampling Population	Sampling Collection	No. of						Score
						Subject	HPV-Infected	HR-HPV	LR-HPV	SI	MI	
1	2011	Yongxi Zhang	Hubei	Female	Exfoliated Cervical Cells	178	68	63	-	-	24	9
2	2011	Yongxi Zhang	Hubei	Female	Exfoliated Cervical Cells	200	73	67	-	-	-	9
3	2012	Yongxi Zhang	Hubei	Female	Exfoliated Cervical Cells	293	130	117	41	-	54	9
4	2013	Xiaofeng Guo	Guangdong	Female	Exfoliated Cervical Cells	166	-	64	-	-	-	9
5	2013	Lili Luo	Hubei, Henan	Female	Exfoliated Cervical Cells	182	67	36	31	44	23	6
6	2013	Yifei Hu	Beijing	MSM	Anal Swab	212	174	-	-	-	-	8
7	2014	Hongyun Zhang	Yunnan	Female	Exfoliated Cervical Cells	301	131	-	-	-	-	7
8	2015	Zhen Li	Shaanxi	MSM	Anal Swab	193	191	183	154	-	-	10
9	2016	Xiangwei Li	Sichuan, Shaanxi, Shanxi	MSM	Anal Swab	104	86	73	-	36	50	5
10	2016	Yu Qin	Yunnan	Female	Exfoliated Cervical Cells	291	-	101	-	-	-	10
11	2017	Qiue Cai	Guangdong	Female	Exfoliated Cervical Cells	206	-	69	-	-	-	9
12	2017	Junxiao Lou	Zhejiang	Female	Exfoliated Cervical Cells	161	-	62	25	-	31	9
13	2017	Jianyu Wan	Guangdong	Female	Exfoliated Cervical Cells	203	-	42	-	-	-	9
14	2018	Lianhua Tang	Guangdong	Female	Exfoliated Cervical Cells	100	55	-	-	-	8	7
15	2019	X. Liu	Zhejiang	Male	Anal Swab	160	82	75	-	-	-	9
16	2019	Qian Wang	Guangxi	Female	Exfoliated Cervical Cells	183	80	-	-	-	-	9
17	2020	Jianyu Wan	Guangdong	Female	Exfoliated Cervical Cells	550	298	194	-	-	122	8
18	2020	Yaping Qiao	Yunnan, Guangxi, Xinjiang	Female	Exfoliated Cervical Cells	695	-	180	-	-	-	10
19	2021	Lihua Fu	Beijing	Female	Exfoliated Cervical Cells	137	52	49	8	-	-	9
20	2021	Hua Meng	Guangxi	Female	Exfoliated Cervical Cells	100	34	-	-	-	-	8
21	2021	Min Feng	Yunnan	Female	Exfoliated Cervical Cells	176	-	59	-	-	-	10
22	2022	Jing Zhang	Zhejiang	MSM	Anal Swab	345	229	-	-	-	-	10
23	2022	Rufei Duan	Yunnan	Female	Exfoliated Cervical Cells	409	140	125	-	-	-	9
24	2022	Rufei Duan	Yunnan	Female	Anal Swab	409	142	124	-	-	-	9
25	2022	Jing Zhang	Zhejiang	Male	Anal Swab	766	373	309	173	-	-	9

MSM: Men Who Have Sex with Men; SI: Single-infection; MI: Multiple-infection.

**Table 2:** Subgroup analysis and meta regression results of HPV infection profiles in PLWH.

Subgroup	No. of			Infection Rate (%)	95% CI (%)	I^2^ (%)	Q_B_	*p* Value	*p* Value of Regression	
	Study	Included	Infection						Univariate	Multivariate
Total	18	4822	2405	52.54	42.11–62.86	97.6				
**Language**							1.43	0.2312		
Chinese	8	1885	933	46.10	38.06–54.24	92.0			Ref	Ref
English	10	2937	1472	57.83	40.46–74.26	98.5			0.2651	0.9971
**Sample**							6.59	0.0102 *		
Anal Swab	7	2189	1277	69.22	48.77–86.38	98.8			Ref	Ref
Exfoliated Cervical Cells	11	2633	1128	41.71	37.41–46.06	83.2			0.0016 *	0.6679
**Gender**							10.34	0.0013 *		
Female	12	3042	1270	41.04	36.96–45.18	84.1			Ref	Ref
Male	6	1780	1135	74.55	54.94–90.05	98.6			<0.0001	0.0174 *

*: *p* ≤ 0.05.

**Table 3:** The characteristics of 34 studies on HPV vaccine acceptability among unvaccinated Chinese people.

ID	Year	First Author	Investigation Form	Sample Source	Investigation Time	Sample Population	No. of			Score
							Effective Questionnaire	Unvaccinated Subjects	Willing to Vaccination	
1	2019	Yafei Li	Offline	Fujian	March to June 2018	Male College Student	1209	1209	465	8
2	2019	Hua Chen	Offline	Sichuan	December 2017	Female	151	144	66	7
3	2019	Yulan Lin	Online	Fujian	13–24 May 2019	Female College Student	997	997	550	6
4	2019	Zhen Li	Offline	Fujian	May 2017 to May 2018	Female	837	837	535	7
5	2019	Yan Ren	Offline	Zhejiang	September 2018 to January 2019	Female	473	473	304	7
6	2020	Xiaoni Wang	Offline	Shaanxi	October to December 2018	College Student	979	979	478	10
7	2020	Chenyu Zhao	Offline	Inner Mongolia	April to May 2019	College Student	1665	1665	558	6
8	2020	Yulan Lin	Online	Fujian	12 May to 8 June 2019	Female	2339	2339	1368	7
9	2020	Si Qin	Offline	Hunan	July to September 2018	Female (20–45)	2101	2101	1230	10
10	2020	Shuai Wang	Online	Multiregion	September to December 2018	Male College Student	1274	1274	493	6
11	2020	Dingyun You	Online	Multiregion	April to September 2019	Female College Student	4220	3757	2010	7
12	2020	Ling Huang	-	Hainan	January 2018 to December 2019	Female	962	910	717	5
13	2021	Hui Chen	Online	Sichuan	February to April 2020	Adult College Student	881	865	568	8
14	2021	Jingjing Deng	Online	Jiangsu	November to December 2019	Female College Student	2169	1996	1755	6
15	2021	Lan Feng	Online	Guangxi	March to April 2020	Female College Student	337	264	150	8
16	2021	RuoSi Wang	Offline	Yunnan	November to December 2019	Female	545	545	424	8
17	2021	Qiyan Yang	-	Hainan	September 2019 to August 2020	Female (20–45)	3476	2850	2089	4
18	2021	Xi Zhang	Online	Multiregion	February to April 2020	Female College Student	975	954	316	8
19	2021	Xiaoxiao Zhang	Offline	Multiregion	January to December 2019	Female College Student	3007	2918	1613	7
20	2021	Wei He	Online	Multiregion	after 2018	MSM	406	406	350	8
21	2021	Shangying Hu	Mix	Multiregion	11-27 April 2019	Adult (18–45)	5000	4888	3740	7
22	2021	Yu Ma	Online	Guangdong	October to December 2018	College Student (20–26)	5307	5143	1156	8
23	2021	Mingyu Si	Online	Multiregion	21 February to 30 April 2020	Female College Student	3867	3765	1208	10
24	2022	Shuyi Hu	Offline	Shanghai	September to December 2020	Female College Student	1992	1825	1452	8
25	2022	Ji Li	Offline	Jiangsu	1-6 December 2020	Female (12–20)	364	312	236	11
26	2022	Jing Shi	Online	Beijing	September to November 2019	Male College Student	935	935	360	8
27	2022	Yinji Wu	Online	Jiangsu	May to June 2021	Adult Male	288	288	140	8
28	2022	Hong Zhang	Offline	Tianjin	April 2021	Male College Student	421	421	99	9
29	2022	Jing Zhang	Offline	Zhejiang	August 2016 to June 2019	Male PLWH	711	711	301	8
30	2022	Zhenwei Dai	Online	Multiregion	after 2016	Adult College Student	7335	7335	4409	8
31	2022	Haiying Pan	Online	Multiregion	June 2021	MSM	889	889	736	7
32	2023	Feng Hu	Online	Heilongjiang	December 2020 to January 2021	Female College Student	312	256	192	8
33	2023	Haiwei Lv	Mix	Shanghai	July to August 2022	Male PLWH	407	340	301	9
34	2023	Luhong Xie	Online	Guizhou	July 2021 to June 2022	Adult College Student	3412	2542	2343	9

## Data Availability

All data used in this study were extracted from published papers and the detail information is attached in the Supplementary Materials.

## Supplementary Materials

The following supporting information can be downloaded at: https://www.mdpi.com/article/10.3390/vaccines11101614/s1.
